# Development of a Low-Cost Portable System for Text-to-Speech Conversion: A Mechanical-Aided Optical Solution for Educational Inclusion

**DOI:** 10.3390/s26175553

**Published:** 2026-09-01

**Authors:** Leonardo Rentería, Margarita Mayacela, Juan Cepeda, Mireya Alvarez, Mario Guillen

**Affiliations:** 1 Facultad de Ingeniería, Universidad Nacional de Chimborazo, Av. Antonio José de Sucre, Riobamba 060108, Ecuador; celia.mayacela@unach.edu.ec (M.M.); jcepeda@unach.edu.ec (J.C.); mdalvarez.fie@unach.edu.ec (M.A.); 2 Carrera de Electrónica, Instituto Superior Tecnológico Tsachila, Santo Domingo 230150, Ecuador; marioguillen@tsachila.edu.ec

**Keywords:** visual impairment, ESP32-CAM, assistive technology, optical character recognition, voice synthesis, inclusive education, distributed architecture, mechanical alignment

## Abstract

This study presents a low-cost assistive reading system based on a distributed architecture, in which an ESP32-CAM performs image acquisition while an Android application executes the image-processing, optical character recognition, and text-to-speech stages. The processing pipeline includes fixed-threshold binarization, morphological dilation, median filtering, Canny edge detection, and projection-based text segmentation. A key contribution is the mechanical sliding rule-frame, designed to maintain horizontal alignment between the camera and the printed text and to reduce perspective-related errors. The system was evaluated through 36 trials conducted with six participants under Low, Medium, and High Lighting conditions. A repeated-measures analysis showed a statistically significant effect of lighting on the Word Recognition Rate (WRR), with significantly lower performance under Low Lighting than under Medium and High Lighting, while no significant difference was found between Medium and High Lighting. Across all trials, the prototype achieved an overall mean WRR of 76.53%, with a median of 78.75% and a maximum observed WRR of 97.50%. Exploratory participant-level correlations between mean WRR, age, and prior reading experience were not statistically significant and were interpreted cautiously because of the small sample size. These findings provide preliminary evidence of the technical feasibility of combining mechanical alignment with a low-cost portable reading architecture for educational accessibility, while highlighting the need for further validation with larger samples and more diverse operating conditions.

## 1. Introduction

Globally, visual impairment represents a critical public health challenge that affects quality of life and socioeconomic inclusion. According to the Global Burden of Disease study, approximately 15% of the population presents visual impairments, with a growing trend over the last three decades [1]. This condition generates disparities in access to health and education, positioning people with disabilities as a vulnerable population in terms of social equity [2]. In this context, the use of body sensors and tracking systems has become essential for monitoring and assisting human mobility [3]. Current research highlights the need for new organizations in experimental designs to address these issues from a scientific and structured perspective [4], always considering the historical evolution of disability and its implications for public health policies [5]. Among technological solutions, Optical Character Recognition (OCR) has emerged as a fundamental tool for interpreting visual information [6]. In Ecuador, the status of rights and access to information for people with disabilities faces significant barriers, especially in the educational field [7]. The advancement of society toward algorithm-based governance poses ethical challenges that must be considered when designing assistive devices [8]. Previous projects have explored the use of deep neural networks for interactive text recognition and speech synthesis [9], supported by the analysis of algorithms and data structures intended to improve processing efficiency [10]. Likewise, the Universal Design for Learning (UDL) approach in the Ecuadorian educational system highlights the urgency of integrating tools that facilitate the effective inclusion of participants with low vision [11]. In the state of the art of wearable devices, emblematic systems such as FingerReader and EyeRing represent milestones in tactile and visual assistance, allowing for sequential text reading through finger-mounted cameras [12,13]. Other developments like HandSight have validated the use of auditory and tactile feedback to guide the user over the paper [14], while wearable biometric technologies have advanced the precision of scanning and human–machine interaction [15]. The core of these systems lies in OCR engines capable of segmenting characters with high fidelity [16]. However, the challenge persists in languages with complex scripts or variations in handwriting, which drives the development of more robust techniques [17]. To improve efficiency on low-cost hardware, the implementation of optimized computer vision algorithms is required. Canny edge detection remains the standard for image segmentation, even in implementations on FPGAs or resource-constrained microcontrollers [18]. Additionally, the use of the Hough Transform allows for identifying the orientation of text lines, improving the legibility of the scanned material [19].

For this reason, this article proposes the design and implementation of a low-cost assistive reading device based on the ESP32 microcontroller [20], intended to support access to printed educational materials for school-aged users with visual impairments. The system adopts a distributed architecture in which the ESP32-CAM performs image acquisition and wireless transmission, while the Android device executes image enhancement, text segmentation, OCR, and speech synthesis. Unlike ring-type devices that depend mainly on free-hand positioning and corrective feedback, the proposed structure incorporates a movable mechanical rule-frame that provides a physical reference for horizontal and vertical displacement. This mechanism is intended to maintain near-horizontal camera alignment, limit uncontrolled rotational and lateral movement, and keep the printed region within the camera field of view during scanning. The image-processing pipeline complements this mechanical guidance by preparing the captured text for segmentation and recognition under the evaluated lighting conditions. Together, these elements form an integrated portable system that combines physical guidance, digital processing, and multimodal interaction to facilitate sequential access to printed text.

## 2. Materials and Methods

This section details the selected hardware architecture, the implementation of the computer vision system for text detection and recognition, and the experimental methodology employed to evaluate the system’s effectiveness.

### 2.1. System Architecture

**Figure 1:**
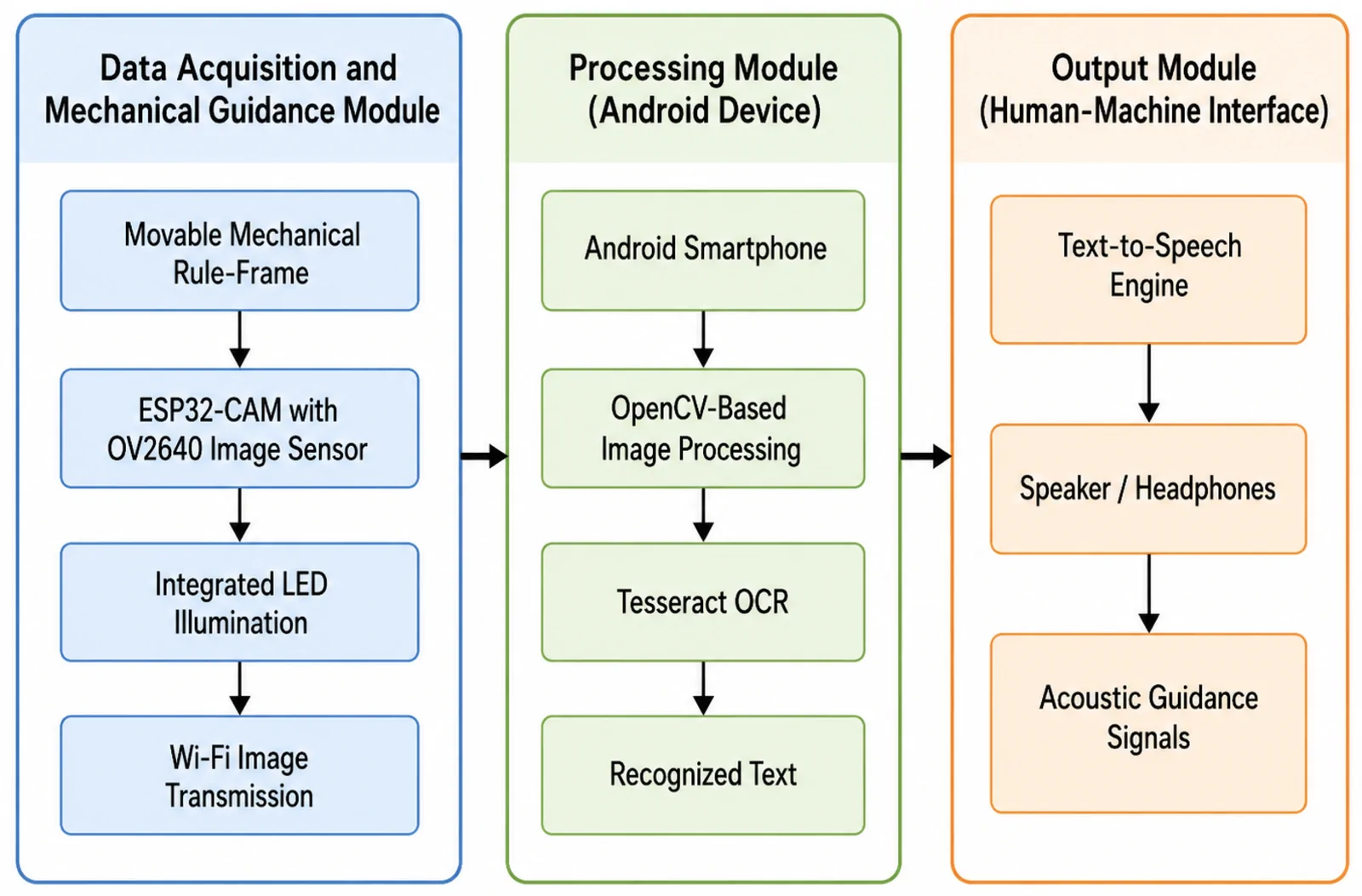
Distributed architecture of the proposed assistive reading system. The mechanical rule-frame supports camera alignment during image acquisition, the ESP32-CAM captures and transmits the image through Wi-Fi, and the Android device performs image processing, OCR, and text-to-speech output.

The assisted reading system was designed under a distributed architecture composed of three main functional blocks (Figure 1): the Data Acquisition Module (Optical System), the Processing Module (Android Device), and the Output Module (Actuators). This modular design leverages the smartphone’s processing power to execute complex computer vision algorithms, while the ESP32-CAM module focuses on image acquisition and data communication.

The distributed architecture assigns each processing task to the component best suited to perform it. The ESP32-CAM is responsible for image capture and wireless transmission, while the Android device handles the more computationally demanding stages, including image preprocessing, segmentation, OCR, and text-to-speech synthesis. This arrangement allows the acquisition module to remain compact and low cost while taking advantage of the greater memory and processing capacity available on the mobile device. The performance of this architecture may also be affected by image quality, Wi-Fi communication stability, and the processing capabilities of the connected Android device, although these aspects were not independently evaluated in the present study.

**Figure 2:**
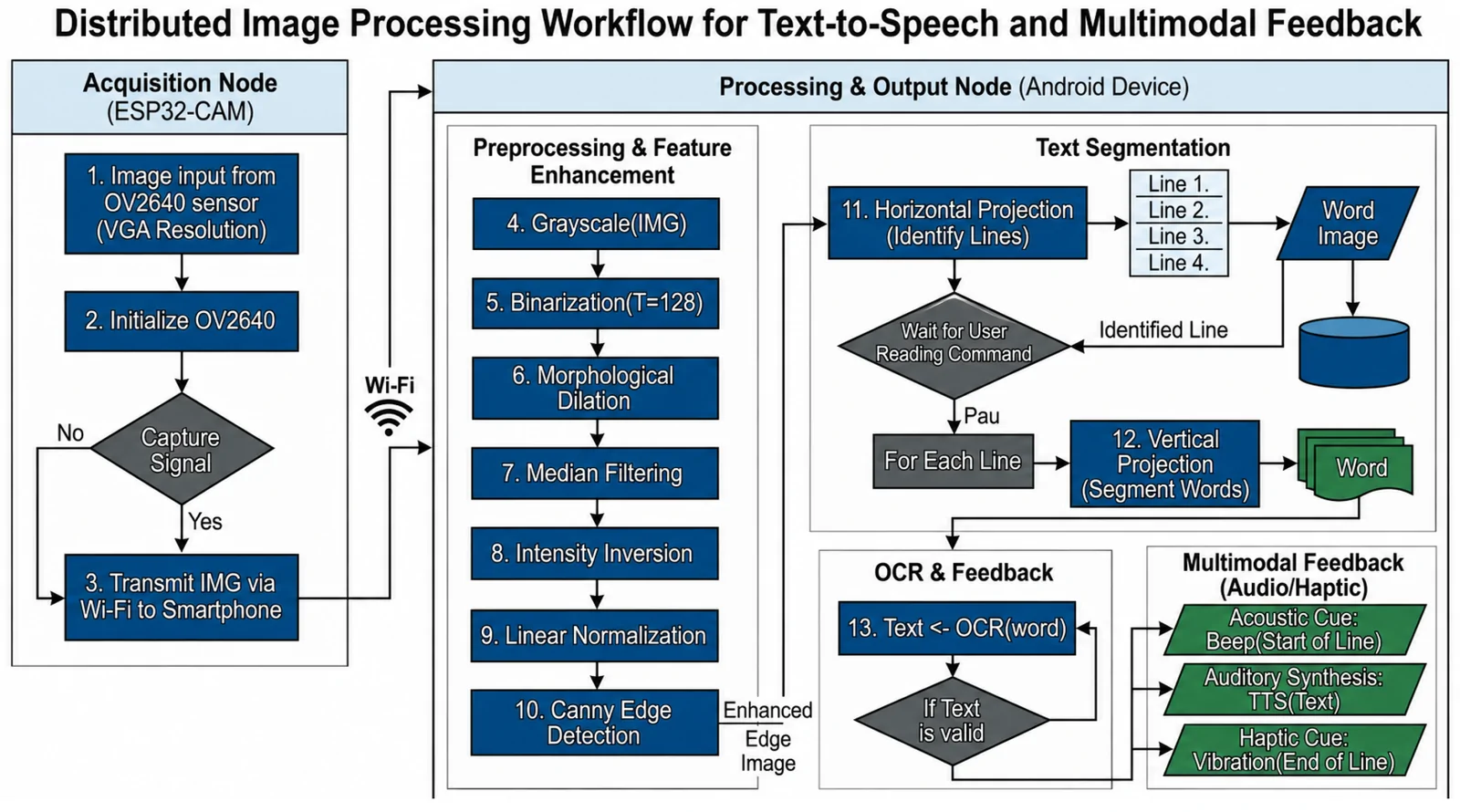
Data flow and digital image processing pipeline for the assistive reading device.

To ensure synchronized operation within this distributed architecture, a specific logical workflow was developed. This process manages the handshake between the ESP32-CAM (Acquisition Node) and the Android device (Processing Node), as well as the sequential execution of the computer vision pipeline. The integration between hardware nodes and software execution is detailed in the following operational flowchart (Figure 2) and Algorithm 1.
**Algorithm 1:** Distributed Text Recognition and Multimodal Feedback Logic**Input:** Image input from OV2640 sensor**Output:** Multimodal feedback (Audio/Haptic)
1:// Block 1: Acquisition Node (ESP32-CAM)2:Initialize OV2640;3:**while** System is ON **do**4:   **if** Capture Trigger received **then**5:     IMG← Capture Frame;6:     Transmit IMG to Android Device via Wi-Fi;7:   **end if**8:**end while**9:// Block 2: Preprocessing and Processing Node (Android Device)10:**while** System is ON **do**11:   Receive IMG from ESP32-CAM Device via Wi-Fi;12:   $IMG_{gray}\leftarrow$ Grayscale(IMG);13:   $IMG_{bin}\leftarrow$ Binarization($IMG_{gray}$);14:   $IMG_{dil}\leftarrow$ MorphologicalDilation($IMG_{bin}$);15:   $IMG_{filt}\leftarrow$ MedianFiltering($IMG_{dil}$);16:   $IMG_{inv}\leftarrow$ IntensityInversion($IMG_{filt}$);17:   $IMG_{norm}\leftarrow$ LinearNormalization($IMG_{inv}$);18:   $IMG_{edges}\leftarrow$ CannyEdgeDetection($IMG_{norm}$);19:   Lines← HorizontalProjection($IMG_{edges}$);20:   Wait for User Reading Command;21:   **for** each line∈Lines **do**22:     Beep(); // Start of line cue23:     Words← VerticalProjection(line);24:     **for** each word∈Words **do**25:        Text← OCR(word);26:        **if** Text is valid **then**27:          TTS(Text);28:        **end if**29:     **end for**30:     Vibration(); // End of line cue31:   **end for**32:**end while**


In this study, the system operates interactively during the user’s reading session. The captured image is transmitted to the Android device, processed, recognized, and converted into speech within the same session, without requiring offline post-processing. No formal latency benchmark was conducted; therefore, the present study does not claim compliance with a predefined temporal-performance threshold.

#### 2.1.1. Data Acquisition Module (Optical System)

**Figure 3:**
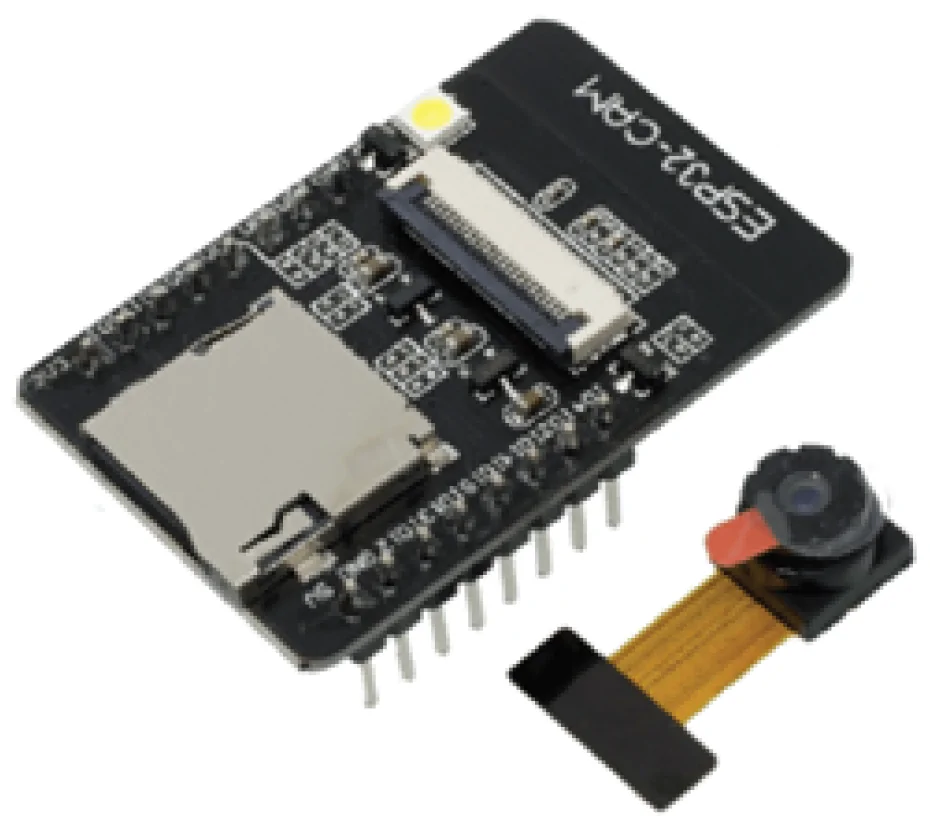
ESP32-CAM module.

This module constitutes the Optical System and serves as the low-cost, portable front-end of the reading system. It is based on the ESP32-CAM module (Figure 3), selected for its ability to integrate a complete image sensing and wireless communication solution in a compact and cost-effective form factor.

The primary components of this module are the Xtensa microcontroller (Espressif Systems, Shanghai, China) and the OV2640 image sensor (Shenzhen Weinan Electronics Co., Ltd., Shenzhen, China), a 2-megapixel CMOS sensor. The acquisition module was configured to capture images at a VGA resolution of 640×480 pixels and a frame rate of 15 fps. The captured images were transmitted through the local Wi-Fi connection to the Android device for subsequent processing [20,21].

Furthermore, the microcontroller (Xtensa) does not perform heavy processing tasks (OCR); instead, it is dedicated to low-level tasks and the communication interface. Its key functions include: initializing and controlling the capture of the OV2640 sensor, managing the image transfer via Wi-Fi to the Android device, and controlling the support illumination LEDs and sound.

#### 2.1.2. Processing Module (Android Device)

The Processing Module was implemented as an Android application responsible for receiving the images transmitted by the ESP32-CAM and executing the image preprocessing, text segmentation, optical character recognition, and text-to-speech stages. This distributed architecture allows the ESP32-CAM to perform image acquisition and wireless transmission, while the Android device provides the computational resources required for the computer vision and OCR operations.

**Figure 4:**
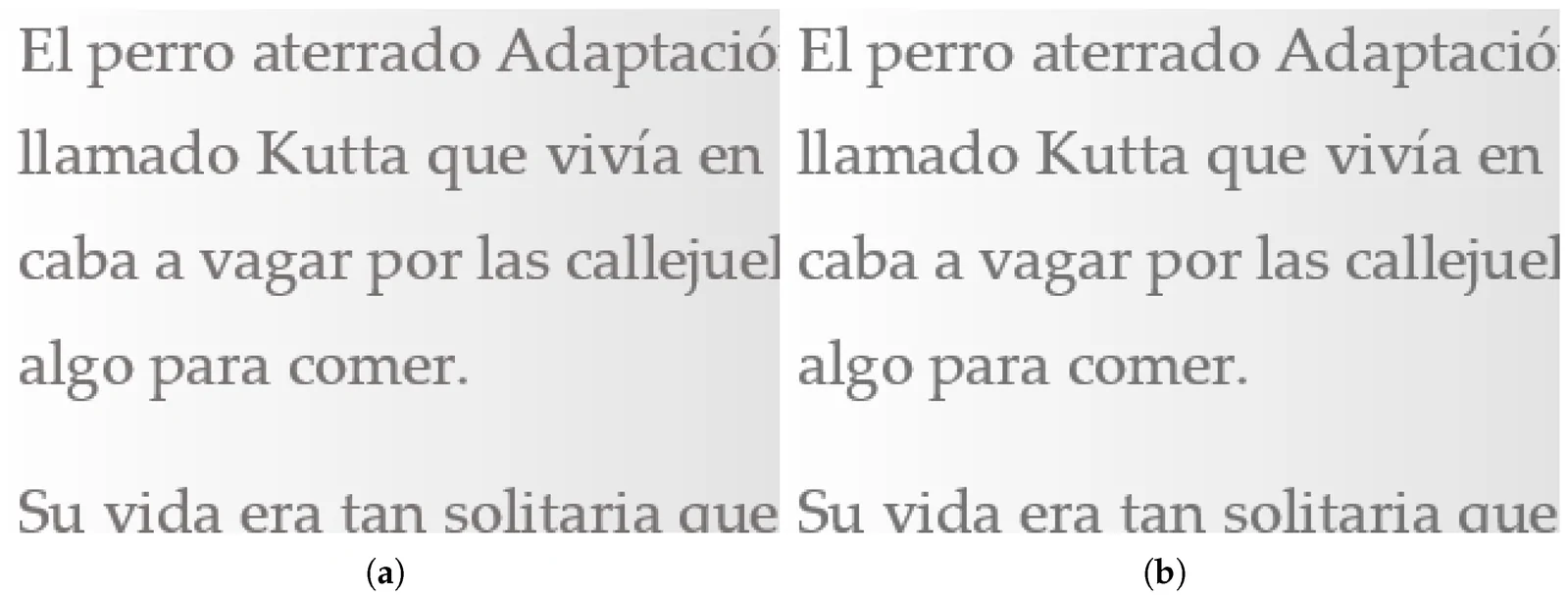
Monochromatic representation using standard luminance weighting: (**a**) original camera-captured region containing a sample of Spanish printed text and (**b**) grayscale conversion of the same region. The partial text shown corresponds to the camera field of view at a given position; as the mechanical rule-frame moves along the page, successive text regions are captured.

Communication between the ESP32-CAM and the Android device was established through a local Wi-Fi connection. The captured images were transmitted to the mobile application at a resolution of 640×480 pixels. All WRR results reported in this study were obtained using Tesseract version 5, configured with the Spanish language model spa.traineddata [22]. The image-processing sequence begins with the original RGB image. To reduce the dimensionality of the visual information while preserving luminance differences between printed characters and the background, the image is converted to grayscale using the standard relationship [23]:

(1) $$I_{g}\left(x,y\right)=0.299\cdot R\left(x,y\right)+0.587\cdot G\left(x,y\right)+0.114\cdot B\left(x,y\right)$$

where *R*, *G*, and *B* represent the intensity values of the red, green, and blue channels, respectively. The weighting coefficients follow the ITU-R BT.601 luminance standard [24]. This transformation preserves the structural information required for the subsequent text-segmentation stages (Figure 4).

The grayscale image was converted into a binary representation to separate the printed text from the page background [25]. A fixed threshold of T=128 was used in the current prototype because of its low computational cost and straightforward implementation. The integrated LED illumination reduced, but did not eliminate, variations in image brightness. The experimental results showed that ambient lighting continued to affect recognition performance, particularly under low-light conditions. Therefore, the fixed-threshold approach should be regarded as an implementation choice for the present prototype rather than as an illumination-invariant solution.

(2) $$I_{b}\left(x,y\right)=\left\{\begin{array}{cc} 1 & \mathrm{if}\;I_{g}\left(x,y\right)>T\\ 0 & \mathrm{otherwise} \end{array}\right.$$

**Figure 5:**
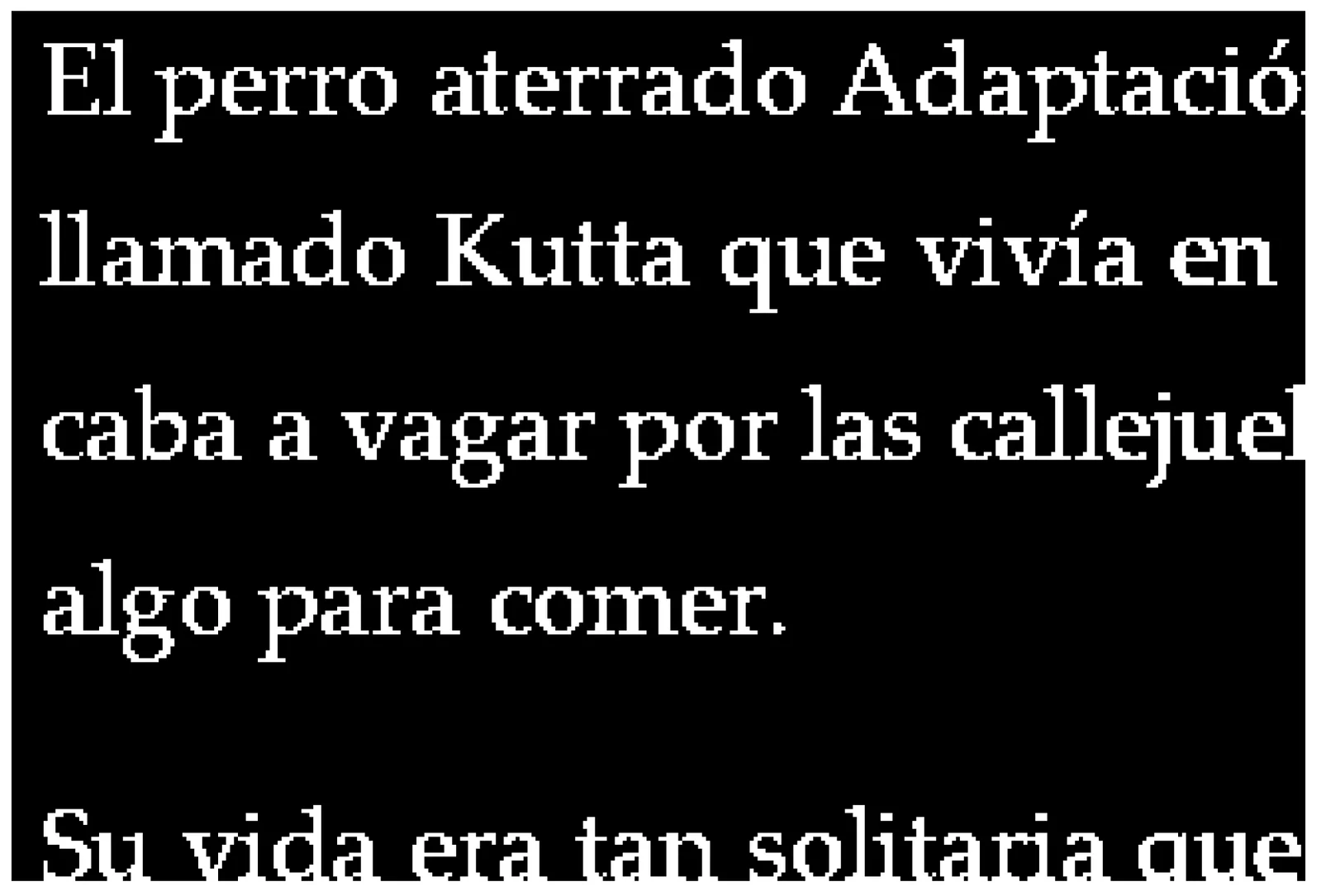
Fixed-threshold binarization using T=128 on the captured Spanish text region.

The binarized image is shown in Figure 5.

**Figure 6:**
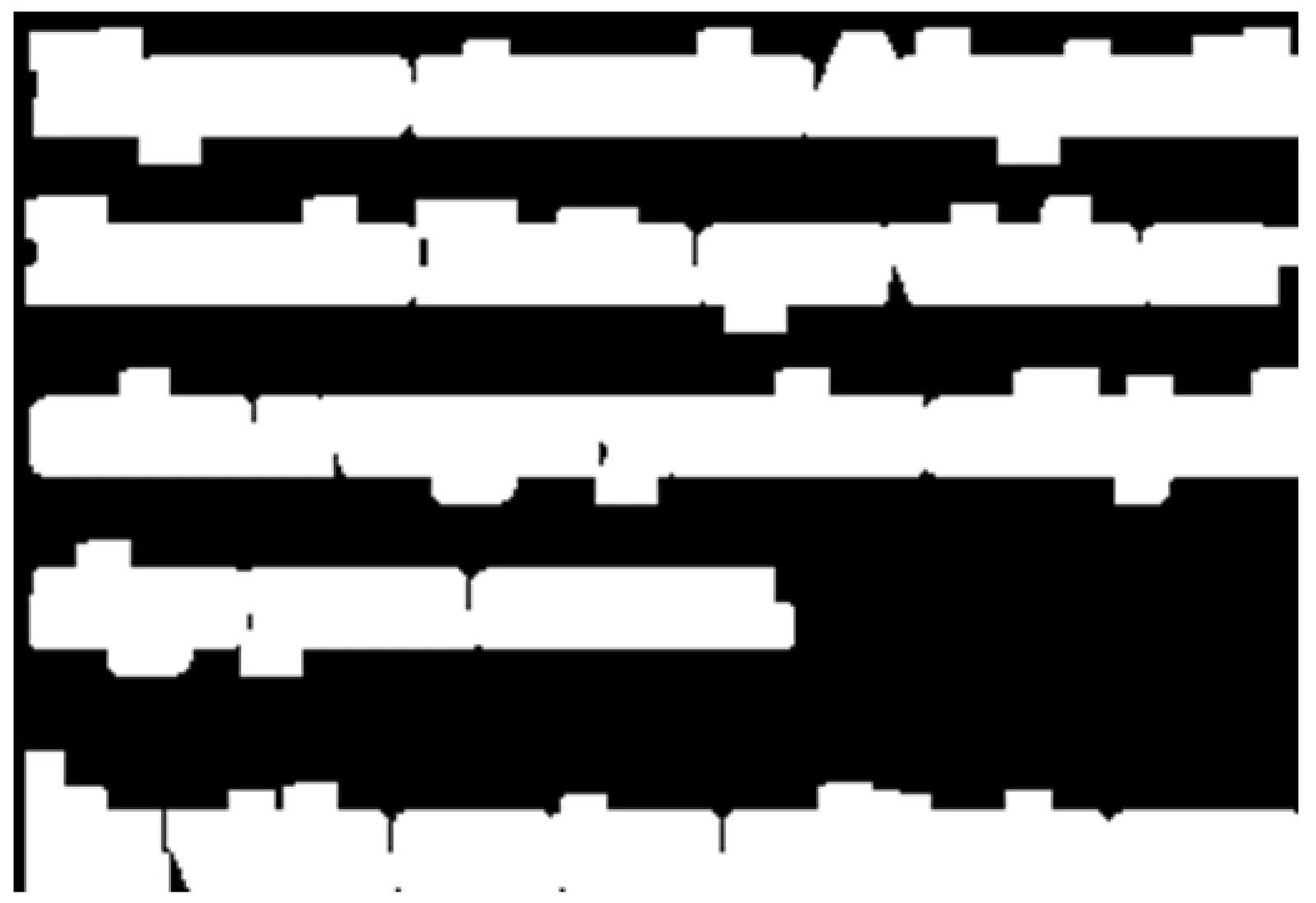
Morphological dilation using a 10×10 square structuring element.

Next, in order to reinforce stroke continuity and connect partially interrupted characters due to the optical limitations of the ESP32-CAM, a morphological dilation is applied using a *S* square structuring element [26]. The operation is expressed as:

(3) $$S={\left[\begin{array}{ccc} 1 & \cdots & 1\\ \vdots & \ddots & \vdots \\ 1 & \cdots & 1 \end{array}\right]}_{10\times 10},\;{{\;\;\;\;\;\;I}_{dil}=I}_{b}⊕S$$

where ⊕ denotes the morphological dilation operation. The kernel size was selected empirically during prototype development. Nevertheless, because a relatively large structuring element may merge neighboring characters or words, its influence is recognized as a limitation and should be examined through a systematic parameter-selection study. The result is shown in Figure 6.

To reduce isolated noise while preserving the structural boundaries of the printed characters, a median filter with a neighborhood radius of K=1 was applied [27]. The effective window size is defined as (2K+1)×(2K+1); therefore, K=1 corresponds to an effective 3×3 filtering window. The filtered value is defined as:

(4) $$I_{\mathrm{med}}\left(x,y\right)=\mathrm{median}{\left\{I_{\mathrm{dil}}\left(i,j\right)\right\}}_{\left(i,j\right)\in N_{3\times 3}\left(x,y\right)}.$$

**Figure 7:**
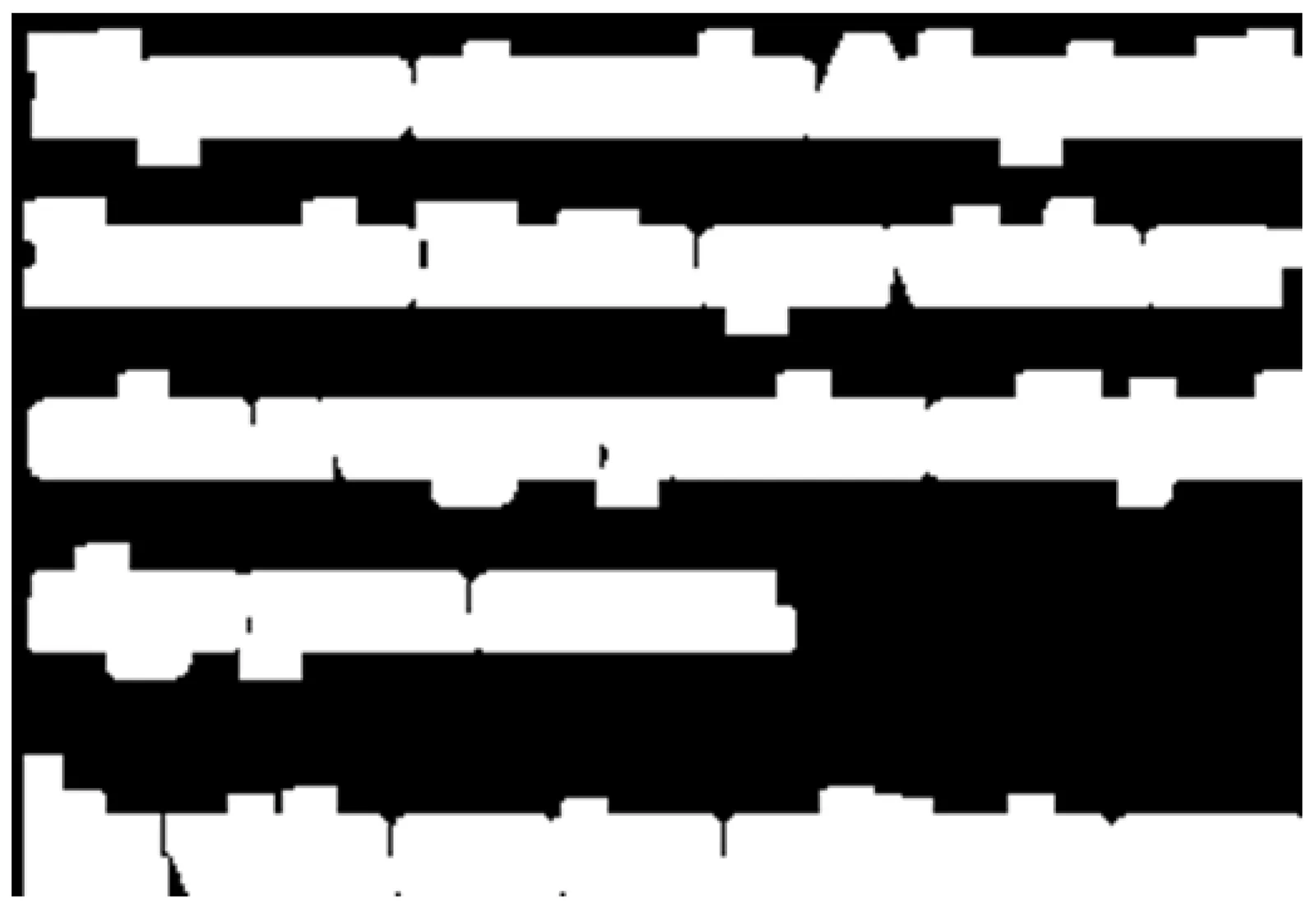
Median filtering using an effective 3×3 window.

The resulting smoothed image is shown in Figure 7.

The filtered image was inverted to obtain the intensity configuration required by the subsequent contrast-adjustment and edge-detection stages. The inversion [28] was performed using:

(5) $$I_{\mathrm{inv}}\left(x,y\right)=255-I_{\mathrm{med}}\left(x,y\right)$$

**Figure 8:**
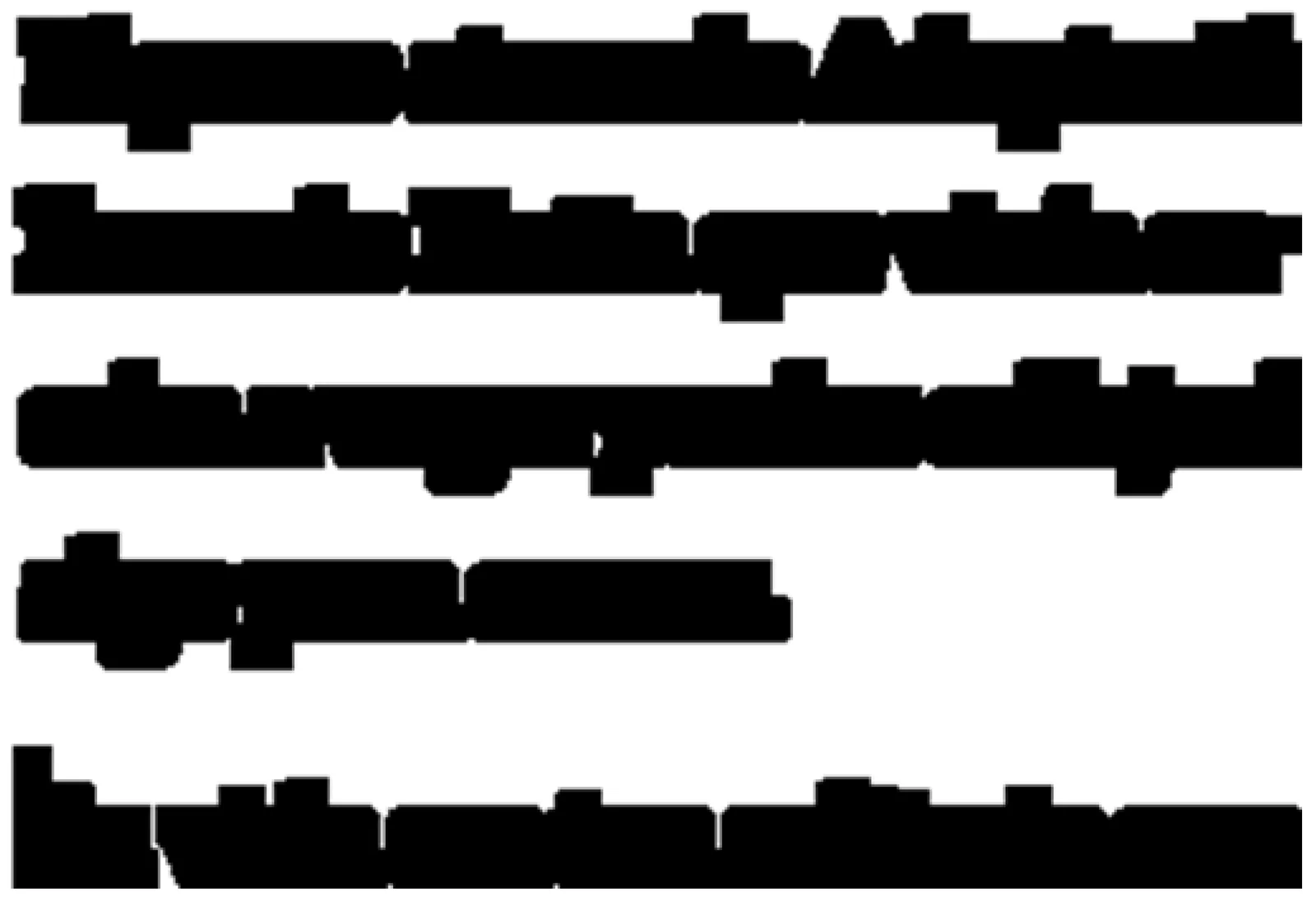
Intensity inversion of the filtered image.

Figure 8 presents the resulting inverted image.

The inverted image is linearly normalized with parameters (α=50,β=100) empirically selected during prototype development to enhance the contrast of the text captured by the OV2640 sensor, generating a suitable dynamic range for edge detection and word segmentation according to the transformation [29]:

(6) $$I_{n}\left(x,y\right)=\alpha \cdot I_{\mathrm{inv}}\left(x,y\right)+\beta$$

**Figure 9:**
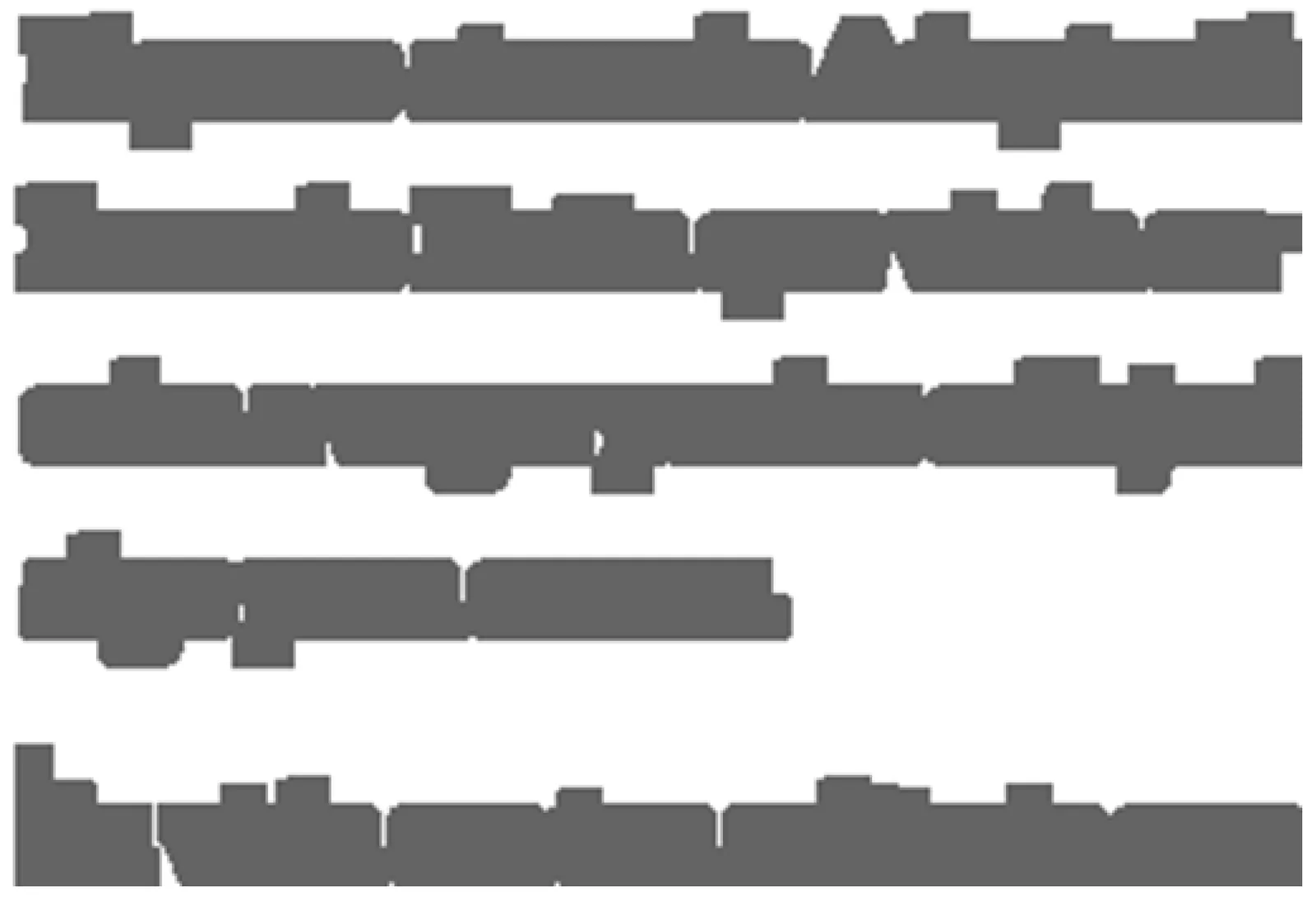
Contrast normalization using the empirically selected parameters (α,β)=(50,100).

The gain factor α amplifies the intensity differences between the printed characters and the page background, while the offset β effectively shifts the histogram into an acceptable dynamic range for the subsequent edge detection. This transformation was used to increase the intensity separation between the text and background before edge detection. The parameters were kept fixed throughout the experiments; however, their influence was not evaluated through a systematic parameter-selection study. Figure 9 illustrates the result of this contrast normalization.

**Figure 10:**
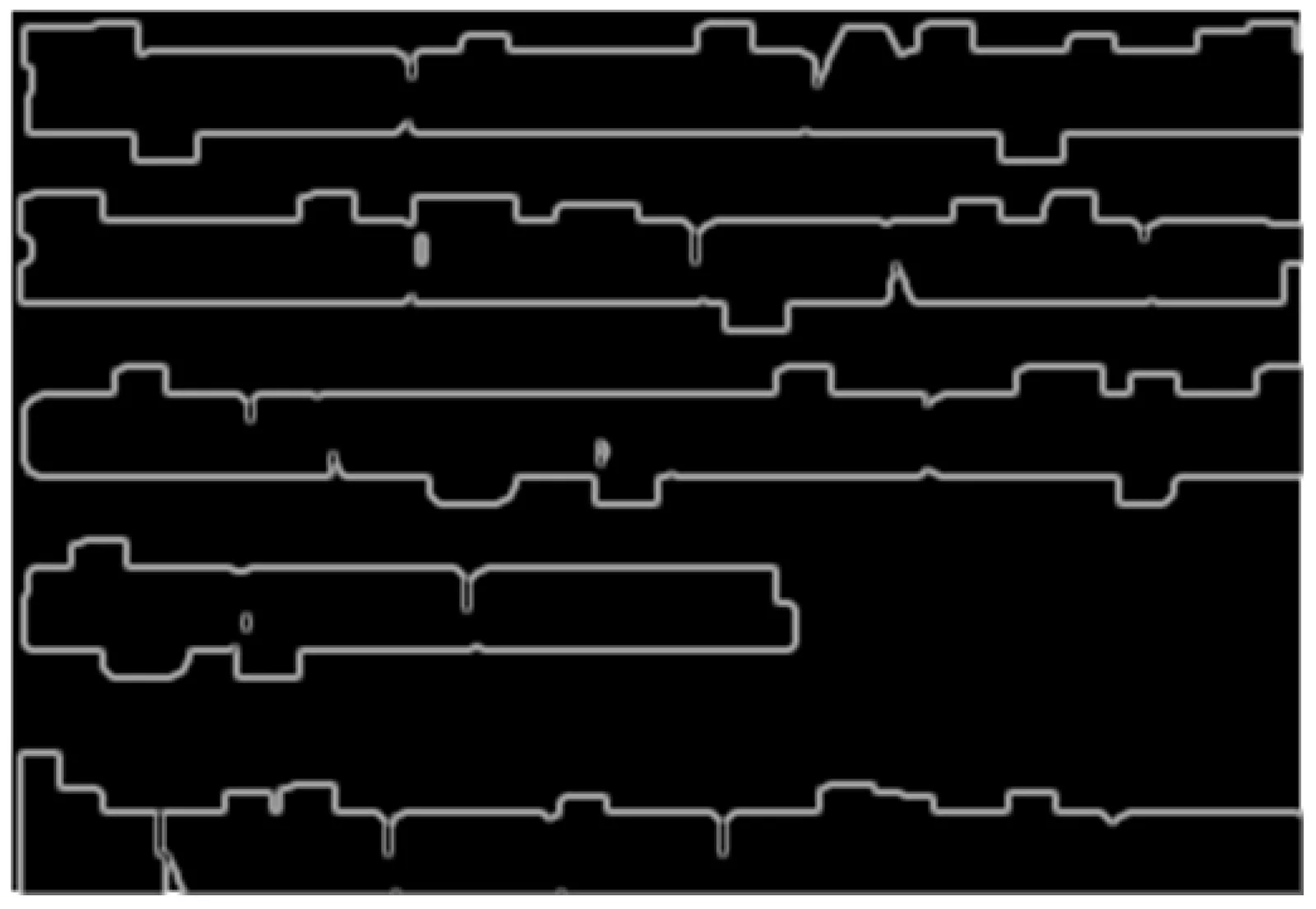
Edge detection using Canny with Sobel kernel and automatic threshold.

To highlight relevant textual contours, the Canny edge detector is employed [30]. Once the image is normalized, the intensity gradient is calculated to identify the structural boundaries of the characters. The gradient is a vector field composed of partial derivatives in the horizontal and vertical directions ($G_{x}$ and $G_{y}$). The gradient magnitude, denoted as G(x,y), is obtained via the Euclidean norm:

(7) $$G\left(x,y\right)=\sqrt{G_{x}{\left(x,y\right)}^{2}+G_{y}{\left(x,y\right)}^{2}}$$

where $G_{x}$ and $G_{y}$ represent the gradient components obtained by applying Sobel operators. This magnitude provides a robust measure of the intensity change rate, allowing the character contours required for precise segmentation to be highlighted. Furthermore, the gradient angle, necessary for the non-maximum suppression step in the Canny algorithm, is calculated as $\theta =\arctan(G_{y}/G_{x})$. The final detected edges are illustrated in Figure 10.

**Figure 11:**
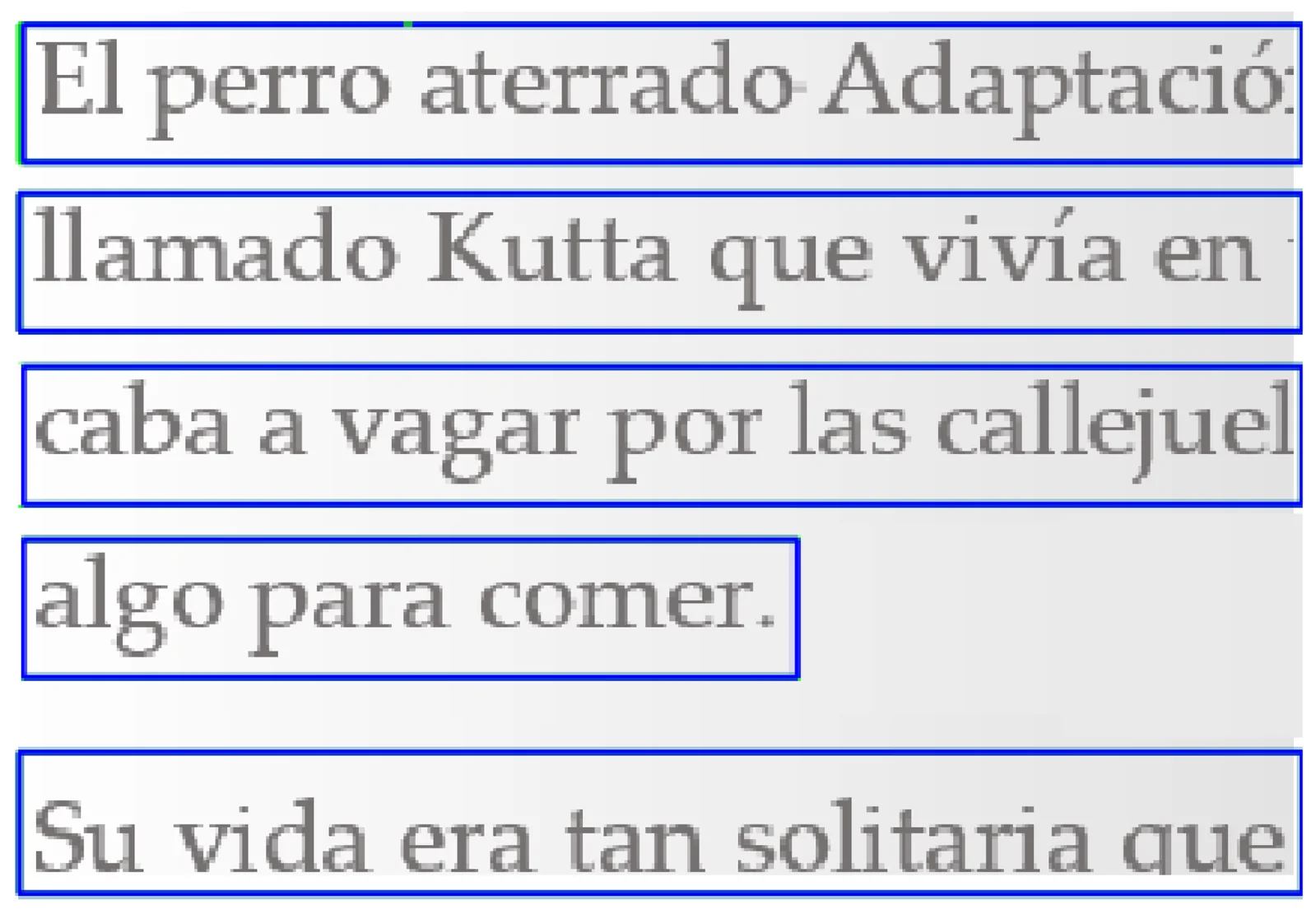
Line segmentation through horizontal projection. Blue boxes indicate the detected text-line boundaries.

Line segmentation is performed through horizontal projection [31], defined by the sum of pixel intensities along the rows:

(8) $$H\left(y\right)=\sum_{x=0}^{W-1}I_{\mathrm{edges}}\left(x,y\right),$$

where *W* is the image width. Consecutive rows with significant projection values were grouped to determine the vertical boundaries of each text line. The resulting segmentation is illustrated in Figure 11.

**Figure 12:**
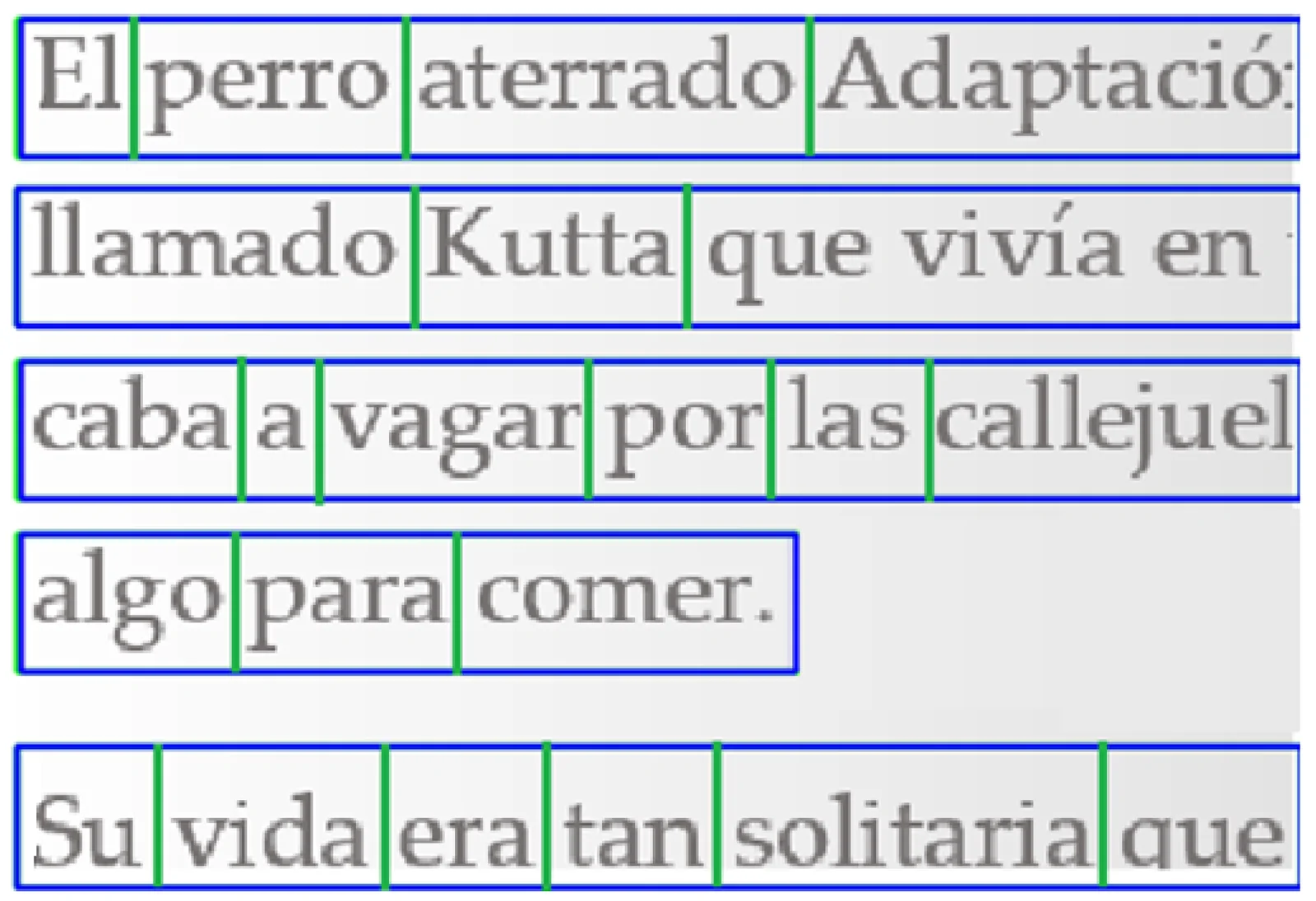
Word segmentation through vertical projection. Blue boxes indicate the detected text-line boundaries, and green vertical lines indicate the detected word-separation boundaries.

Within each detected text line, word segmentation was performed through vertical projection [32]:

(9) $$V\left(x\right)=\sum_{y=y_{1}}^{y_{2}}I_{\mathrm{edges}}\left(x,y\right),$$

where $y_{1}$ and $y_{2}$ denote the vertical bounds of a specific line. This method identifies the gaps between words, as observed in Figure 12.

#### 2.1.3. Output Module

The Output Module is the component responsible for transforming the information processed by the system into auditory and haptic stimuli tailored for users with visual impairments. Its design ensures an accessible, guided, and unambiguous interaction, enabling the sequential reading of the text detected by the processing module.

Once the Android device establishes the local Wi-Fi connection with the ESP32-CAM, the user executes the application again to establish communication with the acquisition module. Upon starting the session, the system verifies the availability of processed text and proceeds to identify the first recognized word. At this point, the system emits an auditory notification instructing the user to press the physical or touch-sensitive button designated to begin reading.

Once the reading mode is activated, the module begins word-by-word audio reproduction, maintaining a continuous and controlled flow. This sequential reading allows the user to perceive each term clearly, avoiding ambiguities or excessive word grouping that could hinder auditory comprehension.

To provide additional reference regarding the text structure, the module integrates haptic and acoustic signals. At the beginning of each line, a brief beep is emitted to indicate the transition to a new sequence of words. Similarly, upon finishing a line, the device generates a short vibration that explicitly communicates the end of that textual line. This combination of multimodal stimuli allows the visually impaired user to identify the document structure without the need for visual feedback, replicating the linear reading experience typical of printed texts.

**Figure 13:**
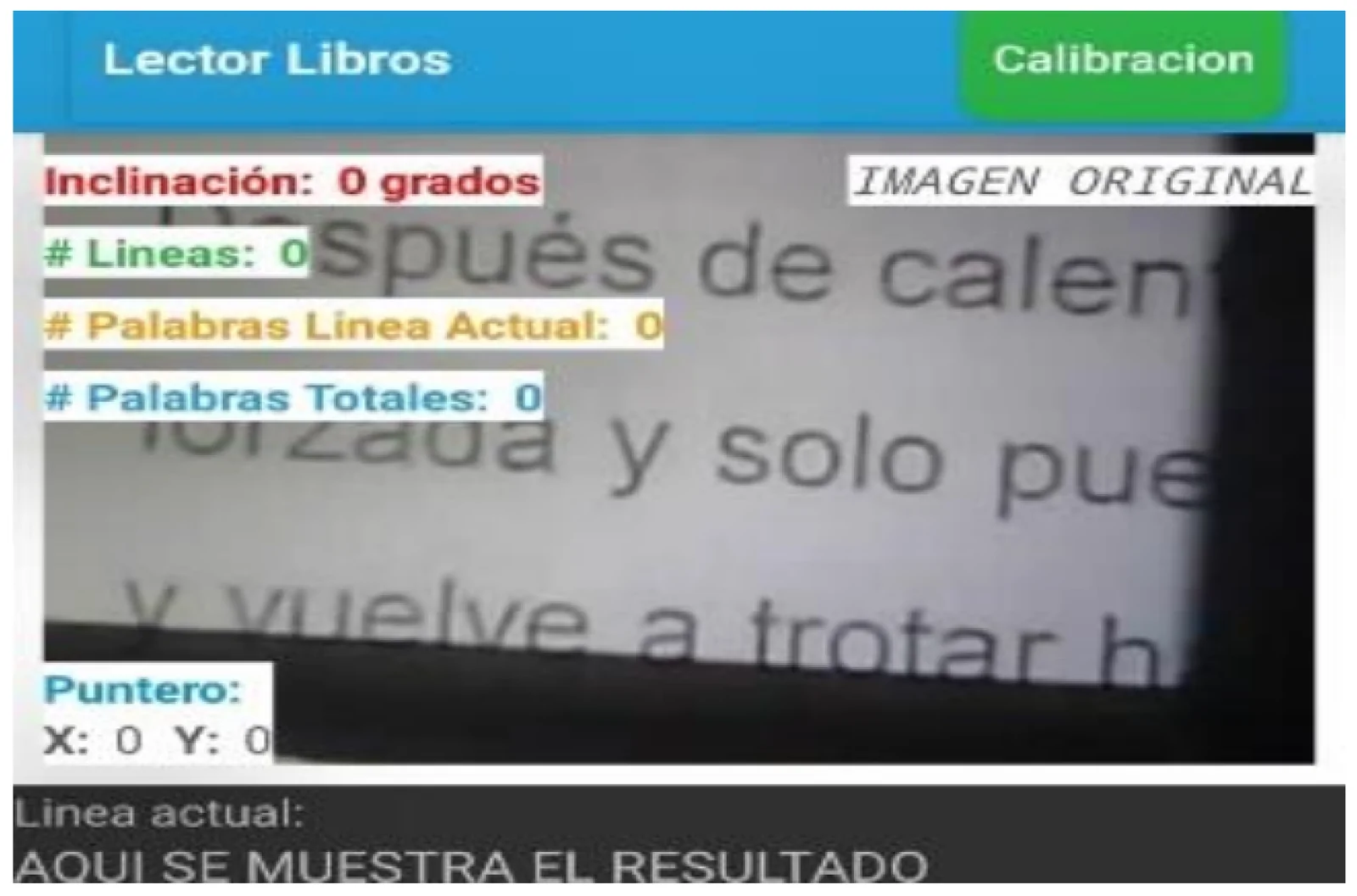
Graphical User Interface (GUI) of the mobile application showing the camera-captured text region and the line- and word-count indicators. The “#” symbol denotes a count, while the colors visually distinguish the different status indicators.

Overall, the Output Module provides a multisensory interface that combines speech output, vibration, and brief auditory cues to indicate the beginning and end of each detected text line, as illustrated in Figure 13. These signals are intended to support sequential navigation through the recognized text.

### 2.2. Hardware

The system hardware consists of two main components: the physical structure and the electronic circuitry, both designed to ensure functionality, usability, and robustness.

#### 2.2.1. Physical Structure

**Figure 14:**
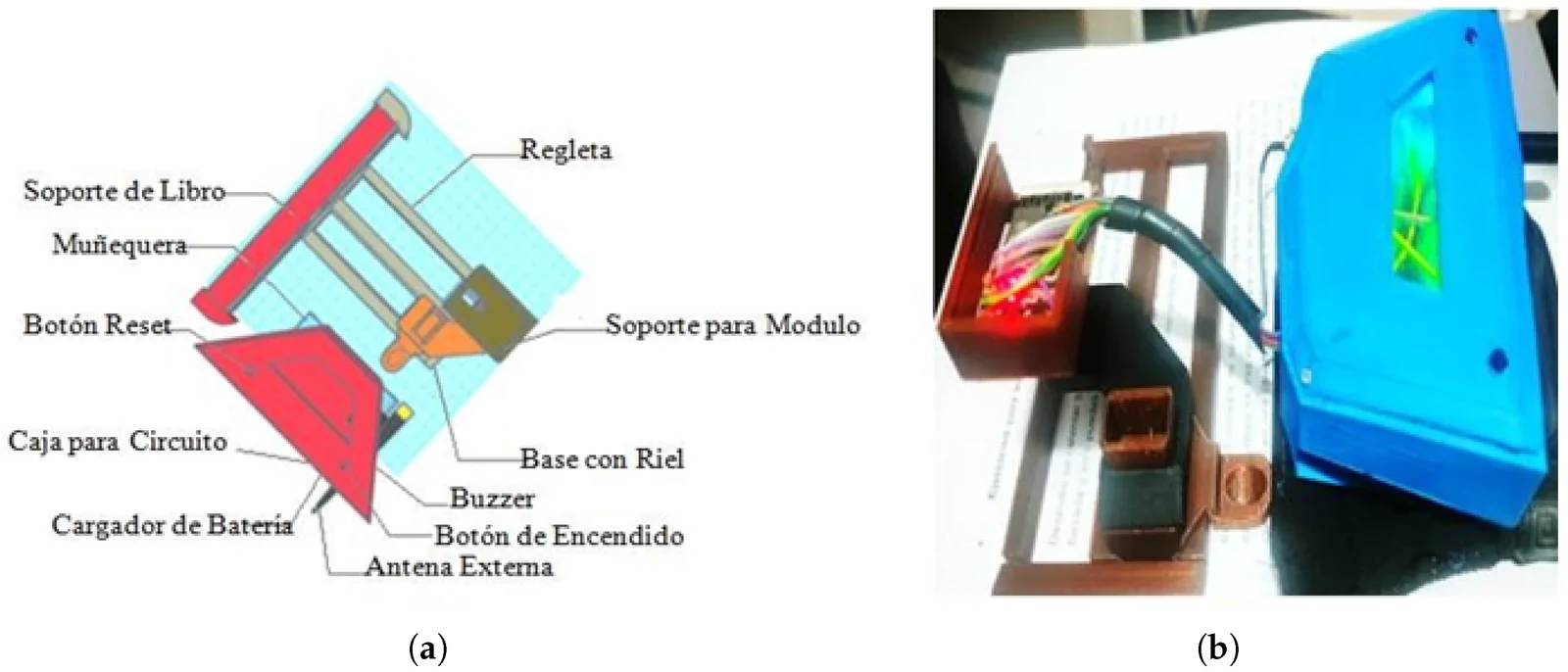
Mechanical design of the reading assistant: (**a**) preliminary 3D CAD design and (**b**) physical prototype of the sliding rule-frame.

The physical structure of the device features a mechanical guide (rule-frame) designed to maintain text linearity during the reading process, facilitating an orderly and precise scan. An integrated rail system allows for smooth and simple displacement of the camera and the processing module, ensuring the device is both ergonomic and user-friendly. Figure 14 illustrates the preliminary design of this structure and presents the final constructed physical prototype.

#### 2.2.2. Electronic Circuitry

**Figure 15:**
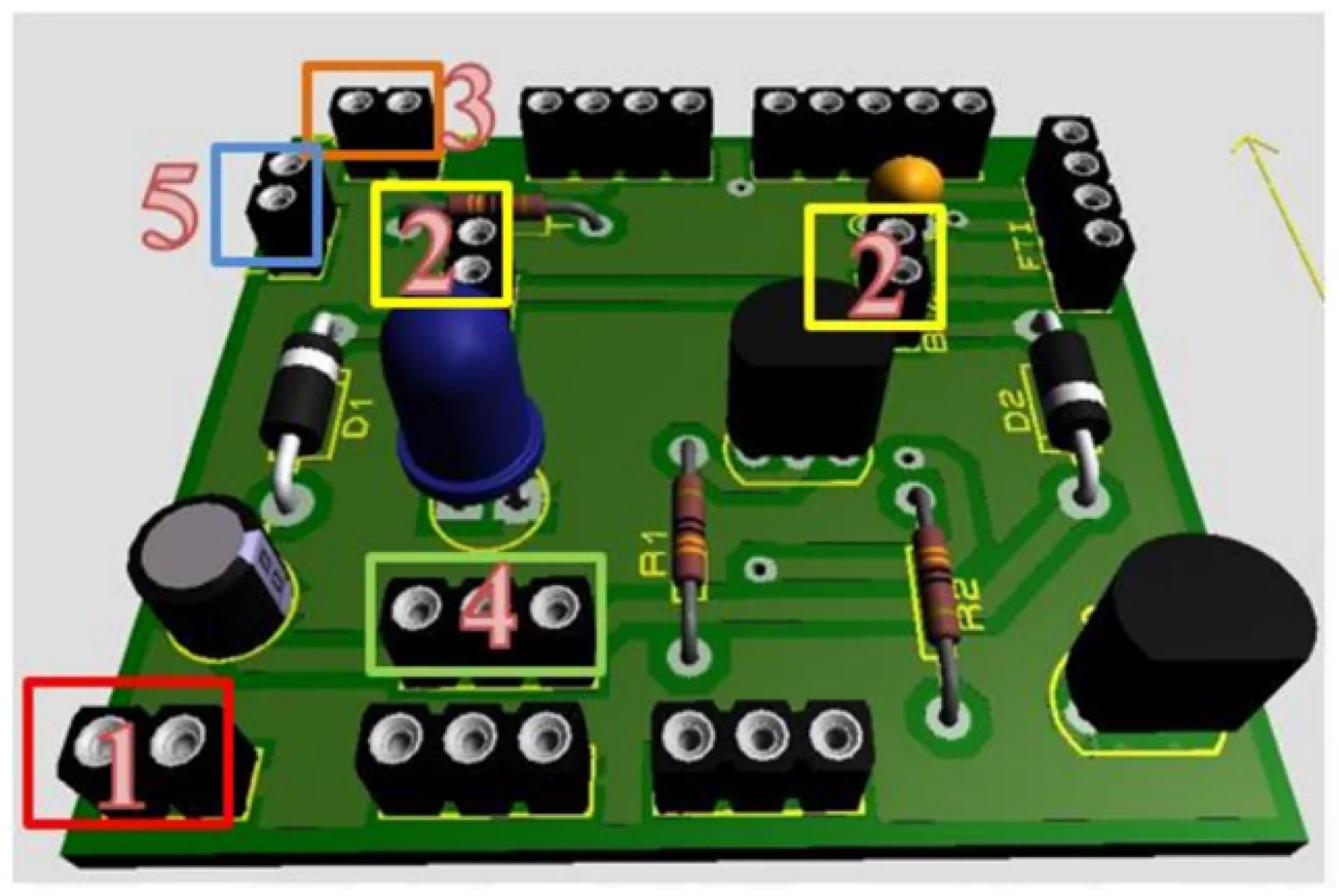
PCB layout and pin configuration for the electronic control system.

The electronic circuit is based on a printed circuit board (PCB) that integrates specific interfaces for system control. Powered by a rechargeable battery to ensure portability, the PCB layout—depicted in Figure 15—features the following functional components:

1. Charging Interface: A Micro-USB Type-B port for battery management and recharging.
2. Feedback Actuators: Dedicated pins for the buzzer and vibration motor, providing acoustic and haptic cues to signal line transitions during the reading process.
3. User Interaction Interface: A push-button terminal for manual control and user-triggered reading initiation.
4. Power Management: An integrated toggle switch for system power state control (On/Off).
5. Status Indicators: An LED interface providing immediate visual feedback on the operational status of the device. These indicators serve a dual purpose: they provide visual status of the power and connectivity state for the primary user’s assistant, and offer diagnostic blink patterns to facilitate system supervision and technical troubleshooting for maintenance personnel.
6. Acquisition Module Interface: A standardized pin header for interfacing with the ESP32-CAM, ensuring reliable image data transmission and system communication.

### 2.3. Experimental Design and Testing Conditions

To evaluate the system’s effectiveness, an experimental study was conducted with six participants using the Word Recognition Rate (WRR) as the primary performance metric. A Spanish text containing 200 words was used in all trials. The device was maintained at a fixed tilt angle of $0^{\circ }$ relative to the text surface, and three lighting conditions were evaluated: Low, Medium, and High.

Each participant completed two trials under each lighting condition. Therefore, each participant performed six trials in total, resulting in 36 experimental trials:

6participants×3lightingconditions×2repetitions=36trials.

The available experimental records confirm that each participant completed two trials under each lighting condition. However, the exact sequence of the lighting conditions and the duration of rest intervals were not systematically recorded. Consequently, potential order, learning, and fatigue effects could not be formally evaluated and are recognized as limitations of the present pilot study.

Because each participant completed repeated trials under the three lighting conditions, the observations were not considered statistically independent. For the inferential analysis, the two repetitions obtained from each participant under each lighting condition were averaged, resulting in one WRR value per participant and lighting condition. The effect of lighting was then evaluated using a one-way repeated-measures ANOVA, with lighting condition as the within-subject factor.

The complete set of raw WRR measurements obtained from the 36 experimental trials is presented in Table 1.

#### 2.3.1. Study Participants

The study population consisted of 6 participants (heterogeneous group including students and homemakers) with varying degrees of visual impairment, ranging in age from 11 to 30 years (Table 2). Informed consent was obtained from all participants or their legal guardians prior to the commencement of the experiments.

#### 2.3.2. Definition and Control of Ambient Lighting

To ensure study replicability, the lighting variable was strictly controlled. Illuminance ($E_{v}$) was measured in lux (lx) using a UT381A digital light meter (UNI-TREND Technology (China) Co., Ltd., Dongguan, China) placed directly on the text surface. Three lighting levels were established to simulate critical environmental conditions, based on the following justifications:

1. **Low Lighting (**$E_{v}<100$ **lx):** Simulates a nocturnal or dimly lit environment. This represents a critical low-contrast condition used to assess the OV2640 sensor sensitivity and the preprocessing algorithm’s capacity to restore image features from dark backgrounds.
2. **Medium Lighting (**$100\;\mathrm{lx}\leq E_{v}\leq 500$ **lx):** Represents the standard illuminance recommended by ergonomic norms (e.g., ISO, IES) for reading tasks. This condition provides a baseline for evaluating device performance under controlled, diffused lighting.
3. **High Lighting (**$E_{v}>500$ **lx):** Simulates bright daylight or high-intensity lighting. This condition evaluates the robustness of the normalization and segmentation algorithms against glare and potential CMOS sensor saturation.

#### 2.3.3. Performance Metrics and Statistical Analysis

1. **Word Recognition Rate (WRR):** WRR was used to assess the accuracy of the optical system and processing pipeline. It is defined as the percentage of words correctly recognized and synthesized relative to the total number of words in the test text, as shown in Equation (10):
  (10) $$\mathrm{WRR}=\left(\frac{W_{\mathrm{correct}}}{W_{\mathrm{total}}}\right)\times 100\%.$$
  where $W_{\mathrm{correct}}$ represents the number of successfully recognized words and $W_{\mathrm{total}}$ is the total word count of the sample text (200 words).
  In the present study, WRR was used as a global recognition metric. It does not distinguish among substitution, deletion, and insertion errors; therefore, the results should not be interpreted as a complete OCR error analysis. Word Error Rate (WER) and Character Error Rate (CER) were not calculated because the experimental records did not preserve the complete recognized-text outputs required for this analysis.
2. **Participant Characteristics:** Age and prior reading experience (RE) were considered in an exploratory participant-level analysis. Information on prior reading experience was self-reported by adult participants and, in the case of minors, was provided by their parents or legal guardians. The responses were coded using an ordinal descriptive scale developed for the present pilot study (Table 3). Because this scale was not independently validated, it was used only for exploratory analysis and not as a diagnostic or predictive instrument.
  The assigned levels represent descriptive categories of reported reading history and should not be interpreted as direct measurements of motor ability, spatial awareness, or visual memory.
3. **Statistical Framework:** Because each participant was evaluated under all three lighting conditions, the effect of lighting on WRR was examined using a one-way repeated-measures ANOVA. The two repetitions obtained from each participant under each lighting condition were averaged before the inferential analysis. Sphericity was assessed using Mauchly’s test. Following a statistically significant overall effect, Bonferroni-adjusted pairwise comparisons were performed. Associations between participant-level mean WRR, age, and prior reading experience were explored using Spearman’s rank correlation. All analyses were conducted using IBM SPSS Statistics, version 23, with a significance level of α=0.05.

## 3. Results and Discussion

The experimental dataset comprised 36 trials obtained from six participants, with two repetitions conducted under each of the three lighting conditions. The global descriptive statistics of the Word Recognition Rate are summarized in Table 4.

Overall, the descriptive results show variability in recognition performance across the experimental trials. The effect of lighting was subsequently examined using a repeated-measures analysis that accounted for the multiple observations obtained from each participant.

### 3.1. Participant-Level Descriptive Summary

To provide a descriptive overview of the variability observed across users, Table 5 summarizes the mean, minimum, and maximum WRR obtained by each participant across the six experimental trials. These values are presented for descriptive purposes only and were not used to establish participant-level differences.

The participant-level summaries show variability in recognition performance across the six trials completed by each user. However, because the study included only six participants and the trials were conducted under different lighting conditions, these descriptive differences should not be interpreted as evidence of individual ability or as generalizable participant effects.

### 3.2. Descriptive Performance and Comparison by Lighting Level

The descriptive analysis showed differences in recognition performance across the three lighting conditions. Table 6 summarizes the mean WRR obtained from the 12 trials conducted under each illuminance level.

The descriptive results indicate lower recognition performance under the Low Lighting condition, whereas similar mean WRR values were observed under Medium and High Lighting. Inferential analysis was subsequently performed to determine whether these differences were statistically significant while accounting for the repeated measurements obtained from the same participants.

#### 3.2.1. Assumption Testing for the Repeated-Measures Analysis

The sphericity assumption was evaluated using Mauchly’s test. The results are presented in Table 7.

Mauchly’s test did not indicate a violation of the sphericity assumption (p=0.066). Therefore, the sphericity-assumed row was used to interpret the repeated-measures ANOVA.

#### 3.2.2. Repeated-Measures Analysis of Lighting Conditions

Because the same participants completed trials under all three lighting conditions, the effect of lighting on WRR was evaluated using a one-way repeated-measures ANOVA. For this analysis, the two repetitions obtained from each participant under each lighting condition were averaged.

The null hypothesis was that the mean WRR did not differ across the three lighting conditions. The results of the repeated-measures ANOVA are presented in Table 8.

The repeated-measures ANOVA showed a statistically significant effect of lighting condition on WRR, F(2,10)=22.21, p<0.001. Therefore, the null hypothesis was rejected, indicating that recognition performance differed across the evaluated lighting conditions after accounting for repeated measurements from the same participants.

#### 3.2.3. Pairwise Comparisons Between Lighting Conditions

Following the significant repeated-measures ANOVA result, Bonferroni-adjusted pairwise comparisons were performed to identify the lighting conditions between which statistically significant differences in WRR occurred. The results are summarized in Table 9.

The Bonferroni-adjusted comparisons showed that WRR under Low Lighting was significantly lower than under Medium Lighting (p=0.025) and High Lighting (p=0.002). No statistically significant difference was observed between Medium and High Lighting (p=1.000). These results indicate that the main reduction in recognition performance occurred under the Low Lighting condition.

### 3.3. Exploratory Participant-Level Analysis

An exploratory participant-level analysis was conducted to examine possible associations between mean WRR, age, and prior reading experience. To avoid treating repeated trials as independent observations, the six WRR values obtained from each participant were averaged, resulting in one mean WRR value per participant. Spearman’s rank correlation was used because of the small sample size and the ordinal and highly unbalanced distribution of the reading-experience variable. These analyses were considered exploratory and were not used to establish predictive relationships. The participant-level data used in this analysis are presented in Table 10.

Spearman’s rank correlation was used to explore possible associations between participant-level mean WRR, age, and prior reading experience. The results are presented in Table 11.

The exploratory analysis showed positive associations between mean WRR and both age and reading experience. However, neither correlation was statistically significant. Therefore, the present data do not provide sufficient evidence to establish age or reading experience as predictors of recognition performance. These findings should be interpreted cautiously because the analysis included only six participants and the reading-experience scores were highly unbalanced.

### 3.4. Distribution and Variability Analysis

To complement the inferential analysis, the distribution of the Word Recognition Rate (WRR) was examined descriptively across lighting conditions. Participant-related variables were analyzed separately at the participant level to avoid treating repeated trials from the same individual as independent observations.

#### WRR Distribution Across Lighting Conditions

**Figure 16:**
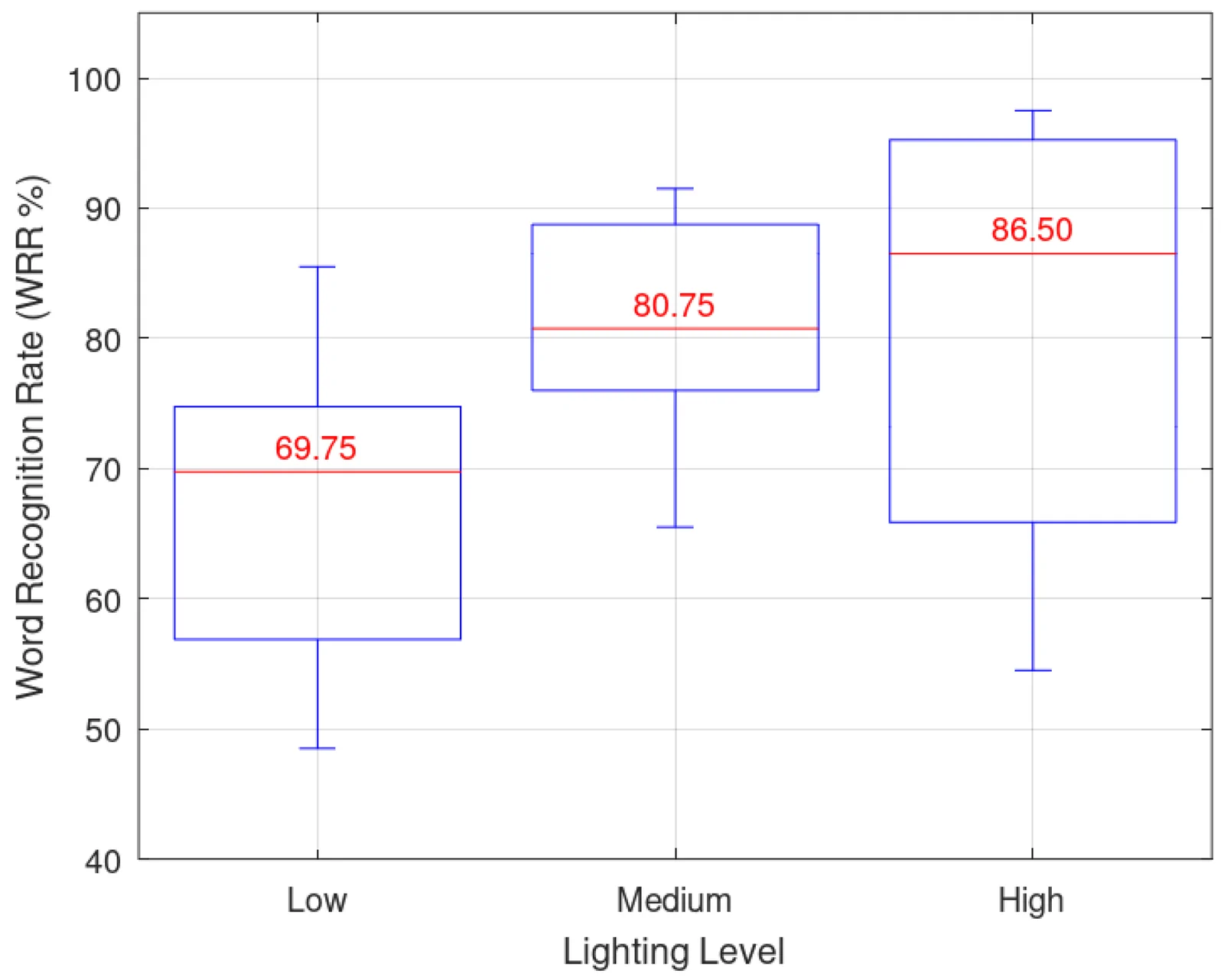
Distribution of Word Recognition Rate (WRR) across Low, Medium, and High Lighting conditions.

Figure 16 shows the distribution of WRR values under Low, Medium, and High Lighting conditions.

Under Low Lighting, the median WRR was 69.75%, whereas under High Lighting it increased to 86.50%. The Low Lighting condition also exhibited greater dispersion than the other two conditions, indicating less consistent recognition performance under insufficient illumination. These descriptive patterns are consistent with the repeated-measures ANOVA, which identified a statistically significant effect of lighting on WRR. Bonferroni-adjusted comparisons showed significantly lower WRR under Low Lighting than under Medium and High Lighting, while no statistically significant difference was found between Medium and High Lighting.

Although the distribution under High Lighting appears more concentrated, this result should not be interpreted as evidence that illumination removes differences in performance across users. Factors related to image acquisition, processing, and user operation may also contribute to the variability observed in WRR. However, the present experiment did not independently quantify camera alignment, motor control, or user skill; therefore, their possible contribution cannot be determined from the available data.

### 3.5. Comparative Analysis and Practical Characteristics

Table 12 presents a contextual comparison between the proposed prototype and three assistive systems reported in the literature: FingerReader [12], EyeRing [13], and HandSight [14]. These systems were developed primarily as research prototypes, and their publications do not report complete commercial prices or detailed bills of materials. Consequently, the cost ranges included in the comparison should be interpreted only as indicative estimates based on currently available prices for the reported components or their closest commercial equivalents.

FingerReader is an index-finger wearable device designed for sequential scanning of printed text. Its hardware incorporates a miniature video camera, two vibration motors for directional haptic feedback, and a dual-material enclosure intended to maintain an approximately fixed camera-to-page distance. Its software stack includes text-extraction algorithms, Tesseract OCR, Flite text-to-speech, and a standalone computer application [12]. The system provides tactile and auditory warnings when the user deviates from the text line; nevertheless, the scanning trajectory continues to depend on the user’s ability to move the finger along the line and correctly interpret the feedback signals.

EyeRing integrates an ATmega32U4 microcontroller, an OV7725 VGA CMOS sensor, an OV529 JPEG compression engine, an RN-42 Bluetooth module, a lithium-polymer battery, and a push-button interface. Captured images are transmitted to an Android smartphone or computer for processing [13]. EyeRing was conceived primarily as a pointing-based interface rather than as a mechanically guided line-scanning system. Consequently, image capture depends on the user correctly directing the finger-mounted camera toward the intended object or printed region. The original study also identified the need for additional feedback to indicate more clearly what the camera was pointing at.

HandSight uses a finger-mounted $1\times 1\;{\mathrm{mm}}^{2}$ AWAIBA NanEye 2C miniature camera capable of capturing 250×250-pixel images at 44 fps [14]. The system provides auditory or haptic directional guidance to assist the user in tracing lines of text. However, its limited camera field of view requires the reader to follow the current line precisely to prevent the text from being cropped or distorted. The reported evaluation identified difficulties associated with changes in camera distance, rapid finger movement, image blur, camera obstruction, loss of position near page margins, and the need to maintain the hand at a specific angle. Some participants also reported discomfort and increased concentration during prolonged use.

These limitations do not imply that the finger-worn systems are ineffective. Rather, they illustrate that auditory and haptic feedback provide corrective information after or while positional deviations occur, whereas they do not physically constrain the trajectory of the camera. In contrast, the proposed prototype incorporates a movable mechanical rule-frame that provides a physical reference for horizontal and vertical displacement. The frame is intended to limit rotational and lateral movement, maintain a more consistent camera-to-text orientation, and reduce dependence on continuous manual correction.

Nevertheless, the present study did not conduct a controlled comparison between the mechanical rule-frame and the guidance mechanisms used by FingerReader, EyeRing, or HandSight. Therefore, the proposed structure should be described as a design response to positioning and alignment difficulties reported in previous research, rather than as proven evidence of superior stability, lower fatigue, or reduced cognitive load.

In comparison with the reference systems, the proposed prototype uses an ESP32-CAM module with an integrated OV2640 camera for image acquisition and Wi-Fi transmission. The Android device performs the main image-processing, OCR, and text-to-speech operations. The experimentally assembled acquisition and mechanical-guidance module had a hardware cost below $70. This amount includes the ESP32-CAM, electronic components, battery, PCB, illumination elements, and mechanical rule-frame, but excludes the Android device.

The recognition results of the proposed prototype were obtained under the experimental protocol described in this study. Across the 36 trials, the system achieved an overall mean WRR of 76.53%, a median WRR of 78.75%, and a maximum observed WRR of 97.50%. These values should not be directly compared with performance values reported for the reference systems because the studies used different datasets, languages, participants, hardware configurations, OCR engines, experimental conditions, and evaluation metrics.

The comparison shows that the proposed rule-frame approaches the positioning problem differently from the reference systems. FingerReader and HandSight guide the user through auditory or haptic feedback when deviations occur, whereas EyeRing depends on free-hand pointing toward the target. In the proposed system, the mechanical structure provides a physical guide for camera movement, helping to maintain alignment before image processing begins. This approach is intended to reduce the need for continuous positional correction during scanning.

However, additional experiments are required to quantify this potential advantage. Future work should compare free-hand and mechanically guided operation using objective measures such as angular deviation, lateral displacement, number of corrective movements, image blur, reading time, recognition accuracy, task completion rate, perceived effort, and user fatigue.

## 4. Conclusions

This study presented a low-cost assistive reading prototype that combines an ESP32-CAM, an Android-based image-processing and text-to-speech pipeline, and a movable mechanical rule-frame designed to maintain horizontal alignment with printed text. The mechanical guidance mechanism represents a distinctive practical feature of the proposed system, as it was incorporated to provide physical support during scanning and to limit alignment and perspective-related variability.

The experimental evaluation included 36 trials performed by six participants under Low, Medium, and High Lighting conditions. The repeated-measures ANOVA showed a statistically significant effect of lighting on WRR. Pairwise comparisons indicated that recognition performance under Low Lighting was significantly lower than under both Medium and High Lighting, whereas no statistically significant difference was observed between Medium and High Lighting. Across all trials, the system achieved an overall mean WRR of 76.53%, a median WRR of 78.75%, and a maximum observed WRR of 97.50%.

The exploratory participant-level analysis did not identify statistically significant associations between mean WRR and either age or prior reading experience. Therefore, the present results do not support considering these variables as predictors of system performance. These findings must be interpreted cautiously because the study included only six participants and the reading-experience scores were unevenly distributed.

The comparison with previous assistive reading devices should not be interpreted as a direct accuracy benchmark because the systems were evaluated using different datasets, languages, participants, OCR engines, and testing conditions. Accordingly, the principal advantage of the proposed prototype lies not in demonstrating superior recognition accuracy, but in integrating a low-cost portable architecture with a mechanical alignment mechanism intended to support more stable interaction with printed text.

Overall, the results provide preliminary evidence of the technical feasibility of the proposed system and its on-the-fly operation during the reading session. However, further validation is required with larger and more diverse participant samples, randomized testing sequences, controlled rest intervals, additional OCR metrics, alternative thresholding methods, parameter-selection experiments, and quantitative measurements of capture, transmission, OCR, and speech-synthesis latency, as well as battery life, power consumption, and communication reliability. These improvements will be necessary to characterize the system’s temporal performance, robustness, and practical usefulness in educational and everyday settings. 

## Figures and Tables

**Table 1:** Detailed raw results of the Word Recognition Rate (WRR) across 36 experimental trials.

Trial	Participant	Lighting	Age	RE	Correct Words	Errors	WRR (%)
1	1	Low	17	0	101	99	50.5
2	2	Low	17	0	97	103	48.5
3	3	Low	11	0	110	90	55.0
4	4	Low	13	2	115	85	57.5
5	5	Low	30	0	120	80	60.0
6	6	Low	26	4	146	54	73.0
7	1	Low	17	0	138	62	69.0
8	2	Low	17	0	145	55	72.5
9	3	Low	11	0	141	59	70.5
10	4	Low	13	2	160	40	80.0
11	5	Low	30	0	163	37	81.5
12	6	Low	26	4	171	29	85.5
13	1	Medium	17	0	149	51	74.5
14	2	Medium	17	0	153	47	76.5
15	3	Medium	11	0	155	45	77.5
16	4	Medium	13	2	146	54	73.0
17	5	Medium	30	0	177	23	88.5
18	6	Medium	26	4	179	21	89.5
19	1	Medium	17	0	163	37	81.5
20	2	Medium	17	0	160	40	80.0
21	3	Medium	11	0	176	24	88.0
22	4	Medium	13	2	179	21	89.5
23	5	Medium	30	0	183	17	91.5
24	6	Medium	26	4	131	69	65.5
25	1	High	17	0	109	91	54.5
26	2	High	17	0	112	88	56.0
27	3	High	11	0	133	67	66.5
28	4	High	13	2	128	72	64.0
29	5	High	30	0	166	34	83.0
30	6	High	26	4	168	32	84.0
31	1	High	17	0	192	8	96.0
32	2	High	17	0	190	10	95.0
33	3	High	11	0	195	5	97.5
34	4	High	13	2	189	11	94.5
35	5	High	30	0	192	8	96.0
36	6	High	26	4	178	22	89.0

**Table 2:** Demographic and clinical characterization of study participants.

Participant ID	Education Level/Occupation	Age (Years)	Disability Level (%)	Observation
Participant 1	12th Grade (High School)	17	80%	Congenital blindness
Participant 2	12th Grade (High School)	17	80%	Congenital blindness
Participant 3	7th Grade (Elementary)	11	84%	Congenital blindness
Participant 4	7th Grade (Elementary)	13	87–100%	Total loss of vision in right eye
Participant 5	Homemaker	30	85%	Congenital blindness
Participant 6	University Graduate	26	95%	Vision loss due to accident (Age 13)

**Table 3:** Descriptive coding of reported prior reading experience used in the exploratory analysis.

Level	Description
0	No reported prior visual reading experience.
1	Limited prior recognition of printed characters or words.
2	Moderate prior experience reading simple printed text.
3	Regular prior experience reading printed materials.
4	Extensive prior experience reading printed materials before vision loss.
5	Extensive prior reading experience and previous use of assistive reading technologies.

**Table 4:** Global descriptive statistics of the Word Recognition Rate across the 36 experimental trials.

Valid Trials	Mean (%)	Median (%)	Standard Deviation	Minimum (%)	Maximum (%)	Range
36	76.53	78.75	14.23	48.50	97.50	49.00

**Table 5:** Descriptive summary of WRR by participant across the six experimental trials.

Participant	Number of Trials	Mean WRR (%)	Minimum WRR (%)	Maximum WRR (%)
1	6	71.00	50.50	96.00
2	6	71.42	48.50	95.00
3	6	75.83	55.00	97.50
4	6	76.42	57.50	94.50
5	6	83.42	60.00	96.00
6	6	81.08	65.50	89.50

**Table 6:** Mean Word Recognition Rate (WRR) by lighting condition.

Lighting Condition	Illuminance Range (lx)	Mean WRR (%)
Low Lighting	$E_{v}<100$	66.96
Medium Lighting	$100\leq E_{v}\leq 500$	81.29
High Lighting	$E_{v}>500$	81.33
**Global Mean**	—	76.53

**Table 7:** Mauchly’s test of sphericity for the within-subject lighting factor.

Within-Subject Effect	Mauchly’s W	Approx. Chi-Square	df	*p*-Value	Conclusion
Lighting	0.257	5.43	2	0.066	Sphericity assumed

**Table 8:** Repeated-measures ANOVA for the effect of lighting condition on Word Recognition Rate.

Effect	SS	df	MS	F	*p*-Value
Lighting condition	824.174	2	412.087	22.211	<0.001
Error (Lighting)	185.535	10	18.553	—	—

**Table 9:** Bonferroni-adjusted pairwise comparisons between lighting conditions.

Comparison	Mean Difference	Standard Error	Adjusted *p*-Value	95% Confidence Interval	Conclusion
Low vs. Medium	−14.333	3.393	0.025	[−26.324,−2.34]	Significant
Low vs. High	−14.375	1.918	0.002	[−21.153,−7.597]	Significant
Medium vs. High	−0.042	1.834	1.000	[−6.524,6.441]	Not significant

**Table 10:** Participant-level data used in the exploratory correlation analysis.

Participant	Age (Years)	Reading Experience	Number of Trials	Mean WRR (%)
1	17	0	6	71.00
2	17	0	6	71.42
3	11	0	6	75.83
4	13	2	6	76.42
5	30	0	6	83.42
6	26	4	6	81.08

**Table 11:** Exploratory Spearman correlations between participant characteristics and mean Word Recognition Rate.

Variable Pair	Spearman’s ρ	*p*-Value	N	Interpretation
Age vs. Mean WRR	0.522	0.288	6	Not significant
Reading Experience vs. Mean WRR	0.439	0.383	6	Not significant
Age vs. Reading Experience	0.103	0.846	6	Not significant

**Table 12:** Contextual comparison of selected assistive reading prototypes and the proposed system.

Feature	FingerReader	EyeRing	HandSight	Proposed Prototype
Development status	Research prototype	Research prototype	Preliminary research prototype	Research prototype
Image acquisition	Miniature finger-mounted video camera	OV7725 VGA sensor with OV529 JPEG engine	NanEye 2C, 250×250 pixels at 44 fps	OV2640 integrated into ESP32-CAM
Processing architecture	PC application with Tesseract OCR and Flite TTS	ATmega32U4, RN-42 Bluetooth, and smartphone or computer	Finger-mounted camera with external processing	ESP32-CAM and Android device
Positioning method	Manual finger scanning with corrective haptic and auditory feedback	Free-hand pointing and image capture	Manual line tracing with haptic or auditory guidance	Horizontal and vertical movement constrained by a mechanical rule-frame
Positioning limitations reported or inferred from the operating method	Requires the user to follow the line and respond to deviation signals	Depends on manual pointing and correct framing of the target	Limited field of view; sensitive to hand angle, distance, speed, and loss of line position	Mechanical frame intended to reduce rotation and lateral displacement
User feedback	Two vibration motors and speech output	Audio or screen output through the connected device	Haptic or auditory directional guidance and speech output	Speech output through the Android device
Indicative component-cost estimate	$30–$50, excluding the computer	$45–$85, excluding the smartphone or computer	$75–$120, excluding the external processing platform	Below $70, excluding the Android device
Evaluation approach	Early feasibility studies	Initial application and user evaluations	iPad-based study with 19 participants and wearable follow-up with four participants	36 trials with six participants under three lighting conditions
Recognition performance	Not directly comparable	Not directly comparable	Not directly comparable	Mean WRR: 76.53%; maximum observed WRR: 97.50%

Note: The limitations included for the reference systems are based on their reported operating procedures and experimental observations. None of the reference systems incorporates a mechanical rule-frame equivalent to the proposed structure. However, the present study did not perform a controlled head-to-head evaluation; therefore, the mechanical frame cannot yet be claimed to provide statistically superior alignment, lower fatigue, or reduced cognitive load. The reported component-cost ranges are indicative estimates and exclude external processing devices, software-development costs, labor, shipping, and taxes.

## Data Availability

The original contributions presented in this study are included in the article. Further inquiries can be directed to the corresponding author.

## Abbreviations

The following abbreviations are used in this manuscript:

WRR	Word Recognition Rate
RE	Reading Experience
OCR	Optical Character Recognition
